# Time Course of Salivary Protein Responses to Cranberry-Derived Polyphenol Exposure as a Function of PROP Taster Status

**DOI:** 10.3390/nu12092878

**Published:** 2020-09-21

**Authors:** Neeta Y. Yousaf, Melania Melis, Mariano Mastinu, Cristina Contini, Tiziana Cabras, Iole Tomassini Barbarossa, Beverly J. Tepper

**Affiliations:** 1 Center for Sensory Sciences & Innovation & Department of Food Science, Rutgers University, New Brunswick, NJ 08901-8520, USA; neeta.yousaf@rutgers.edu; 2 Department of Biomedical Sciences, University of Cagliari, 09042 Monserrato, Italy; melaniamelis@unica.it (M.M.); mariano.mastinu@unica.it (M.M.); tomassin@unica.it (I.T.B.); 3 Department of Life and Environmental Sciences, University of Cagliari, 09042 Monserrato, Italy; c.contini@unica.it (C.C.); tcabras@unica.it (T.C.)

**Keywords:** astringency, salivary proteins, PROP phenotype

## Abstract

Astringency is a complex oral sensation, commonly experienced when dietary polyphenols interact with salivary proteins. Most astringent stimuli alter protein levels, which then require time to be replenished. Although it is standard practice in astringency research to provide breaks in between stimuli, there is limited consensus over the amount of time needed to restore the oral environment to baseline levels. Here we examined salivary protein levels after exposure to 20 mL of a model stimulus (cranberry polyphenol extract, 0.75 g/L CPE) or unsweetened cranberry juice (CJ), over a 10 min period. Whole saliva from healthy subjects (*n* = 60) was collected at baseline and after 5 and 10 min following either stimulus. Five families of proteins: basic proline-rich proteins (bPRPs); acidic proline-rich proteins (aPRPs); histatins; statherin; and S-type cystatins, were analyzed in whole saliva via HPLC-low resolution-ESI-IT-MS, using the area of the extracted ion current (XIC) peaks. Amylase was quantified via immunoblotting. In comparison to baseline (resting), both stimuli led to a rise in levels of aPRPs (*p* < 0.000) at 5 min which remained elevated at 10 min after stimulation. Additionally, an interaction of PROP taster status and time was observed, wherein super-tasters had higher levels of amylase in comparison to non-tasters after stimulation with CJ at both timepoints (*p* = 0.014–0.000). Further, male super-tasters had higher levels of bPRPs at 5 min after stimulation with both CJ and CPE (*p* = 0.015–0.007) in comparison to baseline. These data provide novel findings of interindividual differences in the salivary proteome that may influence the development of astringency and that help inform the design of sensory experiments of astringency.

## 1. Introduction

Astringency is an everyday sensation that is experienced with consumption of polyphenol-rich foods. It is marked by drying, roughing, and puckering of the oral surfaces [1] and is commonly associated with foods such as green tea, coffee, cocoa, berries, and red wine. The sensation of astringency lingers in the mouth with just a single exposure and intensifies with repeated exposure [2,3]. Individual variation in the perception and liking of astringency is well known and could explain large differences in consumer acceptance of polyphenol-rich foods [4,5,6]. Dietary polyphenols can have both desirable and undesirable nutritional effects. Their excessive consumption has been linked with growth inhibition, decreased nutrient absorption and weight loss in animals [7,8,9]. However, due to their immunomodulatory and anti-inflammatory properties, polyphenols have also been shown to promote healthy gut bacteria and reduce risk for cardiovascular diseases, cancer, obesity, and metabolic syndrome [10,11,12]. Despite these health benefits, the astringent, and sometimes bitter, properties of these foods polarize consumers and make it challenging to incorporate polyphenols into everyday diets. Thus, developing a better understanding of the underlying mechanisms of astringency perception and the factors driving consumer hedonics for these foods is important from a public health perspective and could also guide the food industry in formulating new polyphenol-rich foods and beverages that are acceptable to a broad cross section of consumers.

Our understanding of the underlying mechanisms of astringency has evolved over the last 70 years but remains incomplete. Currently, the most widely accepted model for astringency is based on the interaction between polyphenols and salivary proteins. According to this model, when dietary polyphenols first enter the oral cavity, they form hydrogen bonds or hydrophobic interactions with proteins circulating in saliva to form aggregates which grow over time, becoming insoluble precipitates [13,14,15]. These proteins are mainly identified as acidic proline-rich proteins, histatins, cystatins and statherin. Other proteins also participate in this phenomenon, such as mucins, glycosylated and basic proline-rich proteins, which are mostly adsorbed onto oral surfaces and are essential in providing oral lubricity and protecting the salivary pellicle against damage and microbial insult [16,17]. Interaction of polyphenols with these proteins also forms large aggregates, eventually eroding the protective lubricating layers. Together, these actions generate the astringency sensation [18,19], which, in evolutionary terms, might serve as a warning cue against toxicity from over-consumption of these plant materials. A great deal of our understanding of protein–polyphenol interactions in astringency perception has come from in vitro studies [20,21,22,23,24,25,26,27,28,29]. However, in vitro models cannot fully replicate the physiological conditions and dynamics of the human mouth.

The time course of astringency perception and oral recovery are poorly understood. It is known that astringency following oral stimulation takes anywhere from 100 s to 300 s or even longer to recede [1,30,31,32], although the complementary work examining the salivary protein response over time is limited [33]. The intensity of an astringent sensation, its quality (i.e., the predominance of different sub-qualities such as drying, roughing, puckering) and time course depends on the type and concentration of the stimulus used [1,34,35]. Different types of polyphenols (e.g., grape seed tannins, catechins) as well as metal salts and organic acids have been used in astringency studies, but they produce different profiles of astringency perceptions [36,37]. This diversity suggests that there may be more than one mechanism underlying the astringency response. This is underscored by findings that many astringent stimuli may not interact with proteins at all [38]. Astringency has also been described as a tactile or trigeminal response [39], which could involve chemosensation via activation of bitter receptors [40,41]. The use of many different stimuli under varying conditions has led to no general agreement about the length of time necessary to reset the mouth after exposure to an astringent stimulus. Resolving this issue is particularly important for elucidating the mechanisms by which polyphenols interact with specific salivary proteins and for gaining insights into the broad array of other potential mechanisms involved in astringency perception. In the absence of clear consensus on when the oral cavity resets after an astringent exposure, sensory methodology has traditionally varied in the duration of rest breaks—lasting anywhere from a few minutes to even 30 min [42,43].

Superimposed on these uncertainties are individual differences in salivary responses mentioned previously. For instance, Dinnella and colleagues showed that subjects who can maintain relatively constant salivary protein levels after exposure to astringent stimuli experience less astringency [42], whereas the inability to replenish these levels is associated with higher experience of astringency, especially upon repeated sampling [44]. Other work has shown that familiarity, consumption and liking of polyphenol-rich food can impact perception of astringency as well [4]. Genetic variations may be another source of interindividual differences in astringency perception. Variation in the bitterness perception of 6-n-propylthiouracil (PROP) is a general marker for differences in oral sensations [45] and has also been studied in the context of astringency perception [46,47]. Variation in the bitter taste receptor gene, *TAS2R38*, leads to a structural change in the bitter-taste receptor *TAS2R38*, which affects the binding affinity and, as a result, bitterness perception of PROP. For instance, super-tasters can bind PROP strongly and therefore experience the strongest bitterness, while non-taster individuals do not bind PROP [48] and experience none to very little bitterness. Typically, super- and medium-tasters select foods that are less bitter in comparison to non-taster individuals as the former may be more sensitive to oral sensations in wine, fruits and vegetables [46,49,50]. Indeed, studies using bilberry and crowberry juices supplemented with their native polyphenol extracts, have shown that super-tasters (*TAS2R38* homozygous dominant individuals) dislike the more astringent version of these juices [51]. Other work has shown that tasters may consume astringent fruit such as lingonberries less frequently [50].

Melis and colleagues [52] were the first to study the role of PROP taster status in the perception of and salivary response to another astringent fruit, the North American cranberry (*Vaccinium marcoporan*). Cranberries are unique among astringent fruits with the greatest abundance of type-A proanthocyanidins (condensed tannins) [53]. Following oral stimulation with unsweetened cranberry juice, PROP tasters had higher levels of selected salivary proteins (specific subtypes of acidic proline-rich proteins and cystatins) in comparison to non-tasters. Accompanying sensory experiments used tannic acid-supplemented cranberry juice cocktail to modify astringency perception. Cranberry juice cocktail (cranberry juice with added sugar) was used instead of cranberry juice since the latter is routinely disliked by consumers. Results showed that non-taster male subjects perceived less bitterness and astringency from and gave higher liking ratings to tannic-acid supplemented cranberry juice cocktail compared to taster male subjects. These data suggest an important role for PROP taster status and gender in astringency perception and liking although this association has not been observed in all studies [54,55,56,57].

The objectives of the present study were two-fold. First, the study examined the time course of salivary protein response after stimulation with unsweetened cranberry juice (CJ) or cranberry-derived polyphenol extract (CPE). The second objective was to determine interindividual differences in the context of PROP taster status and gender after stimulation with CJ or CPE. We hypothesized that PROP super-tasters will have elevated levels of salivary proteins following stimulation, in line with previous observations [52] and these levels will return to baseline earlier than in non-tasters.

## 2. Materials and Methods

### 2.1. Subject Recruitment

Healthy adults (*n* = 60), between 18–45 years of age were recruited from the Rutgers University community through an email distribution list. Subjects were screened for PROP taste responsiveness; only PROP non-tasters and super-tasters were admitted into the study into groups balanced for gender. Subjects were also screened for general suitability (e.g., demographics, health information) and familiarity with cranberry juice and cranberry products. They had to have consumed such products within the last 2 years. Exclusion criteria included major metabolic diseases (diabetes, kidney disease, etc.), pregnancy, lactation, food allergies, and the use of medications that interfere with taste or smell functions (e.g., steroids, antihistamines, or anti-depressants). Participants who were determined to be medium-tasters for the PROP phenotype were excluded.

The study was approved by the Rutgers University Arts and Sciences Institutional Review Board (Approval#13-309M). All subjects provided written informed consent and were compensated monetarily for their participation.

### 2.2. PROP Taster Status

The participants were screened and classified according to PROP taster status via the paper disk method [58], which has been previously tested for validity and reliability [52,58,59,60,61,62], and strongly correlates with tongue electrophysiological recordings [63,64]. In this procedure, subjects place a filter paper disk impregnated with 1.0 mol/L NaCl (Sodium Chloride, S671-500, Fisher Scientific, Waltham, MA, USA) on the tip of the tongue for 30 s. They rate the taste intensity of the disk using the labeled magnitude scale (LMS), a 100-mm scale anchored with the phrases “barely detectable” to “strongest imaginable.” This procedure is repeated with a second paper disk impregnated with 50 mmol/L PROP (6-n-propyl–2-thiouracil, P3755, Sigma-Aldrich). Subjects rinse with room-temperature spring water before and in between tasting each paper disk. Subjects are classified as non-tasters (NT) if they rate the PROP disk < 15 mm on the LMS; they are categorized as super-tasters (ST) if they rate the PROP disk > 67 on the LMS. All others are classified as medium-tasters. NaCl ratings do not vary with PROP status in this method. Therefore, NaCl ratings are used as a reference standard to clarify the taster status of subjects who give borderline ratings to PROP. This strategy is based on the rationale that non-tasters give higher ratings to NaCl than to PROP, medium tasters give equivalent ratings to both stimuli and super-tasters give higher ratings to PROP than to NaCl.

### 2.3. Test Stimuli

Two oral stimuli were used: cranberry juice (CJ) and cranberry-derived polyphenol extract (CPE). CJ was made from fresh cranberries frozen at −20 °C until use and donated by Ocean Spray Cranberries Cooperative in Chatsworth, NJ. CJ was made in small batches using a standard recipe where 300× *g* of berries were defrosted, washed, and cooked on a stovetop under medium heat for 10 min with 648 mL of spring water. The mixture was filtered through cheesecloth and cooled to room temperature. CPE solution was made using a carrier-free powdered extract (Ocean Spray Cranberries, Inc., Lakeville, MA, USA) added to spring water at a concentration of 0.75 *w/v* g/L. The water was pre-warmed under low heat on a stirring hot plate to facilitate dissolution. Both stimuli were prepared as needed, the day before subject testing, and refrigerated at 4 °C until 30 min before use. The samples were served at room temperature.

### 2.4. Experimental Procedures

#### 2.4.1. Overall Study Design

Subjects participated in two test sessions. They were instructed to refrain from consuming astringent foods for approximately 8 h prior to each session. A list of such foods was provided to them. They were also prohibited from eating, drinking (except plain water), chewing gum, using breath mints, mouthwash or brushing their teeth for 2 h prior to the test sessions. In addition, they were asked to refrain from consuming any alcoholic beverages for 24 h prior to each session.

During session 1, subjects were familiarized with the procedures and completed demographic questionnaires. During session 2, subjects provided saliva samples by spitting directly into a plastic polypropylene cup, had been refrigerated at 4 °C until ready to be used. First, resting saliva was collected over 1 min to enable the measurement of baseline salivary protein levels. After a 5 min rest period, subjects were given 20 mL of one of the two astringent stimuli (either CJ or CPE), asked to swish the sample in their mouth and then swallow it completely. Subjects then provided saliva at 5 and 10 min after swallowing.

**Figure 1:**
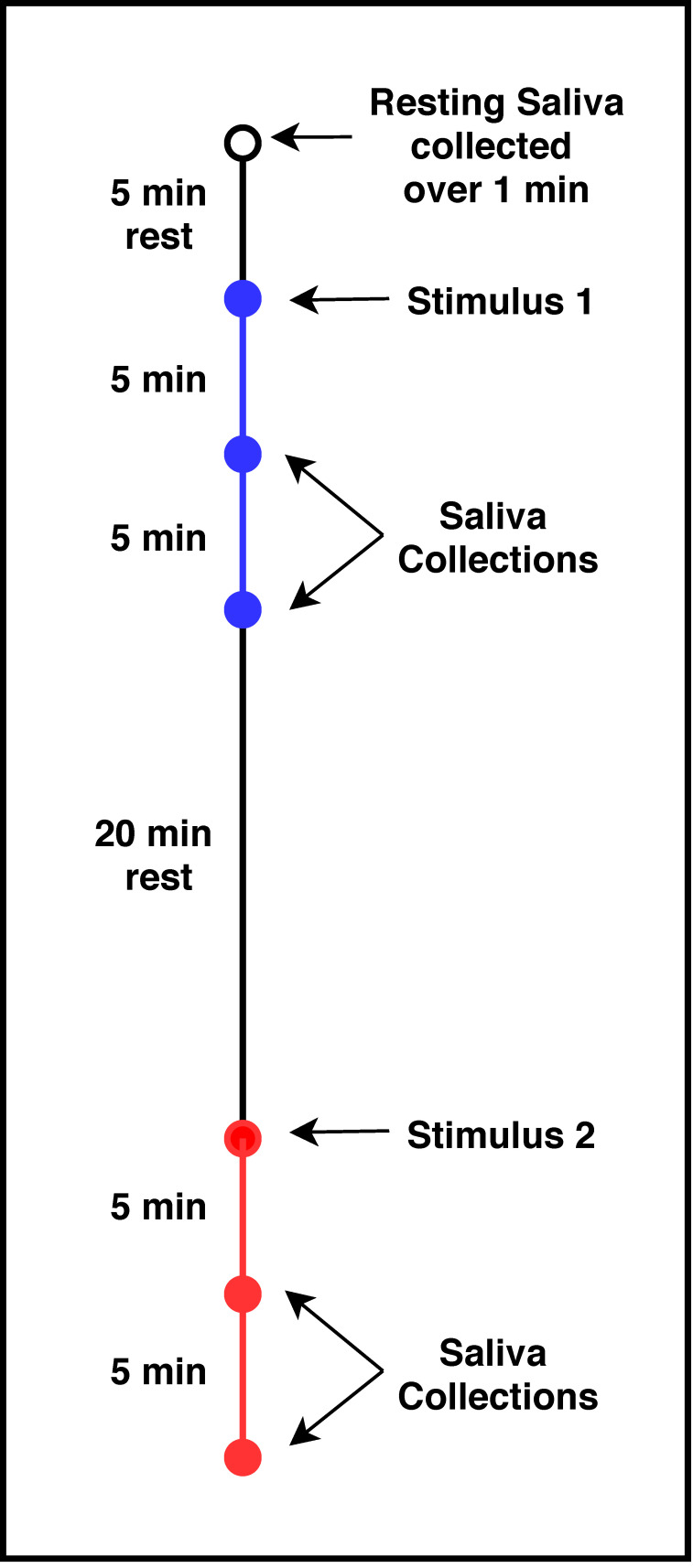
Saliva collection timeline.

After a 20 min break, the subject was provided with the second astringent stimulus and followed the same procedure for saliva collection as above. As a result of this procedure, there were five saliva collections, a resting sample, and saliva collected at 5 and 10 min after exposure to each stimulus (CJ and CPE). The order of presentation (CJ first or CPE first) was randomized across subjects (Figure 1).

#### 2.4.2. Saliva Treatment

Each saliva sample was transferred into two microcentrifuge tubes, which had been maintained on ice (0.5 mL per tube); one tube was prepared for High Performance Liquid Chromatography-low resolution-Electrospray Ionization-Ion Trap-Mass Spectrometry (HPLC-ESI-MS) analysis while the other was prepared for immunoblot procedure.

For the HPLC-ESI-MS analysis, 0.2% trifluoroacetic acid (TFA, Sigma-Aldrich, St. Louis, MO, USA) was added to the saliva in a 1:1 *v/v* ratio. These samples were centrifuged at 8000× *g* at 4 °C for 15 min. The supernatant was separated from the pellet and stored at −80 °C until chromatographic analysis. For the immunoblot procedure, a protease inhibitor cocktail solution [mix of 1 tablet/1.4 mL of cOmplete^®^ Protease Inhibitor Cocktail (Roche Diagnostics, Indianapolis, IN, USA) and 175 mM NH_4_HCO_3_ (Ammonium Bicarbonate, Sigma-Aldrich, St. Louis, MO, USA)] was added to saliva in a 1:2 *v/v* ratio. These samples were stored at −80 °C until Dot-blot immunoblot analysis.

#### 2.4.3. Total Protein Content Quantification

Bicinchoninic Acid (BCA) Protein Assay Kit (Sigma-Aldrich, St. Louis, MO, USA) was used to quantify total protein content of the saliva samples according to manufacturer’s instructions. The total concentration was used to normalize protein levels for dot blot analysis.

#### 2.4.4. Alpha Amylase Analysis

##### Alpha Amylase Purification

To purify amylase protein, a volume of 6 mL of whole saliva was collected and treated with 0.2% TFA, as previously described. The sample was injected in an Ultimate 3000 Micro HPLC apparatus (Dionex, Sunnyvale, CA, USA) equipped with a photodiode detector (UV-VIS) and the chromatographic column was a Vydac-C8 with 5 µm particle diameter (column dimensions 250 × 10 mm) (Hesperia, CA, USA).

The following solutions were utilized for purification: 0.06% (*v/v*) aqueous TFA (eluent A) and 0.05% (*v/v*) TFA in acetonitrile-water 80/20 (eluent B) with flow rate at 2.8 mL/min. Salivary proteins were eluted using a linear gradient from 0% to 60% of B in 40 min, and from 60% to 100% of B in 5 min. Protein detection was carried out at a wavelength of 214 nm. The total injected saliva volume was 800 μL. Collected salivary fractions were analyzed in HPLC-ESI-MS and the fraction containing amylase was lyophilized and then dissolved in 0.1% *v/v* TFA for a total volume of 500 µL. BCA assay was then performed, as previously described, to determine the concentration of amylase in the resulting solution.

##### Alpha Amylase Quantification

The concentration of alpha amylase in the salivary samples (individual samples, un-pooled) was estimated semi-quantitatively by using dot-blot technique, where the protein samples were spotted directly onto a PVDF membrane (0.2 µm pore size; Immun-Blot^®^ PVDF Membrane, Bio-Rad Laboratories, Inc., Italy). To set up a dot blot assay, the saliva samples treated with cOmplete protease inhibitor cocktail were first diluted with Tris Buffered Saline (TBS: 20 mM Tris-HCl pH 7.6, 150 mM NaCl) so that each diluted sample would have the same amount of total protein content (adjusted to 0.38 μg/μL). Before transferring the samples on to the PVDF membrane, it was pre-wetted with methanol for 1 min, then transferred to TBS for 2 min.

The amylase fraction, purified from whole saliva, was used as a standard in 4 concentrations (0.01, 0.02, 0.05, 0.1 µg/µL). All samples and standards were spotted onto the wet PVDF membrane in triplicate. Specifically, each test sample was spotted in a volume of 1µL (0.38 µg/µL of total protein content). The membrane was blocked with blocking agent 5% of BSA (Bovine serum albumin, Sigma Aldrich) in TBS-T buffer (20 mM Tris-HCl pH 7.6, 150 mM NaCl, 0.05% Tween 20) for 1 h at room temperature. Subsequently, the membrane was incubated with primary antibody (dilution 1:1000; Amylase G-10: sc-46657-Santa Cruz Biotechnology, Inc.) in 5% of BSA in TBS-T buffer, for 1 h. Three washes for 5 min with TBS-T buffer were performed and the membrane was incubated for 1 h with secondary antibody (dilution 1:5000; Rabbit anti-Mouse IgG, Secondary Antibody, HRP ThermoFisher Scientific). After three further washes with TBS-T, the membrane was incubated for 5 min with ECL substrate (Clarity Western ECL Substrate, Bio-Rad, Laboratories, Inc, Italy) for fluorescence signal development and captured on the Chemidoc MP Imaging System (Bio-Rad, Hercules, CA, USA). Analysis of images obtained were performed using Image Lab 6.0.1 software (Bio-Rad Laboratories Inc., Hercules, CA, USA). Signals of samples were determined and shown as intensity values which were transformed by the software with volume tools in value of concentration (µg/µL) for each sample by using the standards as references. Each sample was analyzed in triplicate with acceptable coefficient of variation (CV%) set as below 15%.

#### 2.4.5. HPLC-Low Resolution-ESI-IT-MS Analysis

Supplementary Table S1 shows the salivary proteins and peptides analyzed in each of the salivary samples (individual samples, un-pooled) collected using the HPLC-low resolution-ESI-IT-MS technique according to [65]. 30 μL of the acidic soluble fraction corresponding to 15 μL of whole saliva was used (1:1 *v/v* dilution). Only proteins/peptides characterized in human saliva by applying the same analytical conditions in previous studies [66,67] were analyzed in the present investigation. Average mass values (Mav), obtained by deconvolution of averaged ESI-MS spectra automatically performed by using MagTran 1.0 software [68], and elution times of proteins/peptides were compared with those determined under the same experimental conditions in our previous studies [66,67].

Experimental Mav were also compared with the theoretical ones available at the UniProt-KB human data-bank (http://us.expasy.org/tools). The quantification of each protein and peptide was based on the area of the HPLC-ESI-IT-MS extracted ion current (XIC) peaks. The XIC analysis reveals the peak associated with the peptide of interest by searching, along the total ion current chromatographic profile, the specific multi-charged ions generated by the protein. The area of the ion current peak is proportional to concentration, and under constant conditions it may be used to perform relative quantification of the same analyte in different samples [69].

### 2.5. Statistical Analyses

Statistical analyses of the protein data were performed with XLSTAT Statistical and Data Analysis Solution (Addinsoft 2020, New York, NY, USA) and SAS 9.4 Analytical Software (Cary, NC, USA). Normality testing was done using the Anderson–Darling test while homogeneity of variances was tested using Bartlett’s test. For non-normal distributions, data were transformed using the Johnson transformation.

To determine potential effects of stimulus order presentation (CJ first or CPE first) on alpha-amylase as well as protein families (totals of basic proline-rich proteins (bPRPs), acidic proline-rich proteins (aPRPs), statherin, histatin and S-type cystatins), a repeated measures ANOVA (between subjects factor: order; within-subjects factor: treatment, order*treatment) was used.

Transformed data were then analyzed using a repeated measures ANOVA with PROP taster status and gender as between-subjects factors to determine inter-individual differences. In early analyses, we included the effects of ethnicity in our models, but finding no effets of this variable, it was dropped from subsequent analyses. Post-hoc comparisons were performed with Bonferroni’s method.

None of the transformations improved normality for the individual proteins. In these cases, Friedman’s ANOVAs (non-parametric test) were performed on the untransformed data to understand the effect of stimulation on the individual protein levels. These analyses were conducted on individual bPRPs (P-F, P-J, P-D, P-H, IB-8a Tot., II-2 Tot., IB-1 Tot. and Ps-1), aPRPs (PC, PRP-1, PRP-3), histatins (Hist 1 and Hist 5 and 6), S-type cystatins (Cyst S, Cyst S1, Cyst S2, Cyst SN and Cyst SA) and PB. Finally, post-hoc comparisons for the individual proteins (17 in all) were done following Bonferroni corrections (*p* = 0.05/17 = 0.002) to adjust for the large number of comparisons.

To understand variation in individual protein levels with respect to PROP taster status and gender, multiple Kolomogrov–Smirnov pairwise comparisons were performed at each time point. Specifically, cumulative distribution frequency curves of non-taster subjects were compared to those of super-taster subjects at resting, 5 and 10 min after CJ and CPE exposure. Similarly, comparisons were made for gender differences at each of the time points. However, this approach did not reveal significant effects of PROP taster status and gender at the individual protein level. Therefore, these data are not reported.

Data reported in the figures are means (±SEM) and show original, untransformed values.

## 3. Results

Table 1 shows subject characteristics. The subject pool (*n* = 60) was mostly Caucasians (*n* = 43) and the rest were Asians (*n* = 17). Taster x gender subgroups were approximately balanced with 25.0% female non-tasters (*n* = 15), 26.7% female super-tasters (*n* = 16), 23.3% male non-tasters (*n* = 14) and 25.0% male super-tasters (*n* = 15). Mean participant age was 22.0 ± 0.6 years.

### 3.1. Effect of Stimulation on Protein Response

#### 3.1.1. Protein Families

*Overall order effects:* There was no effect of stimulus presentation order on levels of total aPRPs, bPRPs, statherins, histatins and S-type cystatins over the time course (*p* = 0.976–0.211, not significant).

**Figure 2:**
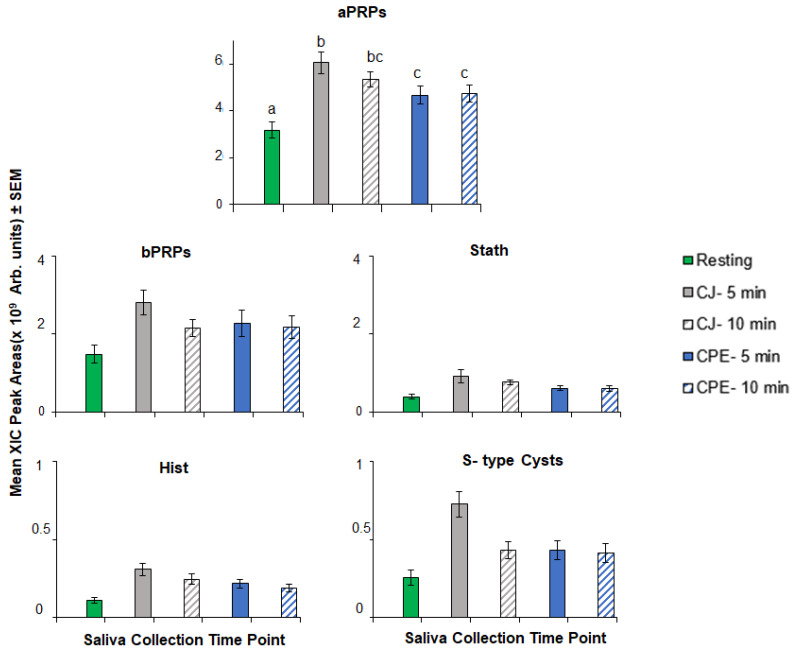
Extracted ion current (XIC) peak areas (mean values × 10^9^ ± SEM) in arbitrary units for salivary protein families following stimulation with cranberry juice (CJ) and cranberry-derived polyphenol extract (CPE) (*n* = 60). Values with different superscript letters (a, b etc.) differ at *p* < 0.05.

*Overall treatment effects:* The effects of stimulation with CJ and CPE on protein families are shown in Figure 2. There was a general trend of higher levels after stimulation with both CJ and CPE. However, this effect was only statistically significant for aPRPs (*F*_[4,225]_ = 24.96, *p* < 0.000). aPRP levels were higher relative to baseline at 5 min and remained elevated at 10 min after stimulation with CJ (*p* < 0.000). The same pattern was observed after stimulation with CPE; levels were higher in comparison to baseline at 5 min (*p* < 0.000) and remained elevated at 10 min after stimulation (*p* < 0.000). No significant main effects of stimulation were observed on other protein families (bPRPs, statherins, histatins and S-type cystatins).

**Figure 3:**
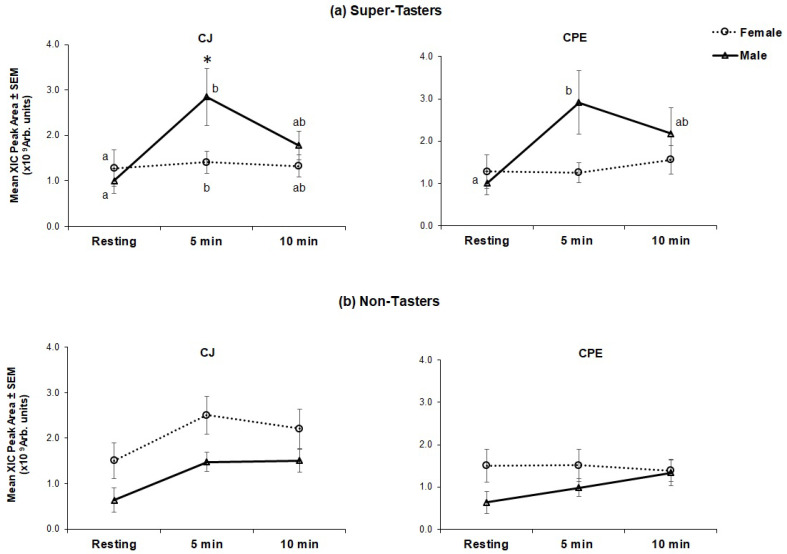
Effect of stimulation on total bPRP (basic proline-rich protein) levels in taster by gender subgroups. Mean extracted ion current (XIC) peak areas ± SEM (×10^9^ arbitrary units) are shown for super-taster (**a**) and non-taster subjects (**b**). Left panels show response to stimulation to CJ, while right panels show response to CPE. The same resting values were used for CJ and CPE. Superscripts (^a,b^) show significant differences in protein levels across the time course in each subgroup (male ST, female ST, male NT or female NT) and * shows differences between males and females at each time point. Non-taster females (*n* = 15), non-taster males (*n* = 14), super-taster females (*n* = 16) and super-taster males (*n* = 15).

*Interaction effects:* There was no overall effect of stimulation on bPRP levels (with either CJ or CPE). However, there was a significant gender*taster*treatment effect (*F*_[4,224]_ = 2.60, *p* = 0.037) on bPRPs (Figure 3).

Separate analyses were conducted in non-taster and super-taster groups. There were significant differences in bPRP levels across the time course in super-taster subjects with CJ (*F*_[4,116]_ = 3.20, *p* = 0.015) (Figure 3a). Super-taster males had higher levels at 5 min than they had at resting, and at 10 min these levels declined to intermediate values, which were not statistically different from levels at either resting or at 5 min. Female super-tasters showed a statistically significant, but small increase at 5 min, that was not considered to be physiologically meaningful. In comparisons by gender, male super-tasters had higher peak bPRP levels (at 5 min) in comparison to female super-tasters (*p* = 0.018). Male super-tasters showed the same pattern of response to CPE as they did for CJ; levels of bPRPs were higher at 5 min after stimulation (*p* = 0.007) compared to resting and declined to intermediate levels at 10 min. Female super-tasters did not show any effect of stimulation to CPE, and there were no differences between genders at any time point.

Within non-tasters (Figure 3b), females generally had higher levels of bPRPs at resting as compared to males (non-significant). No effect of PROP taster status was seen after stimulation with either CJ or CPE among non-tasters.

#### 3.1.2. Individual Proteins

**Figure 4:**
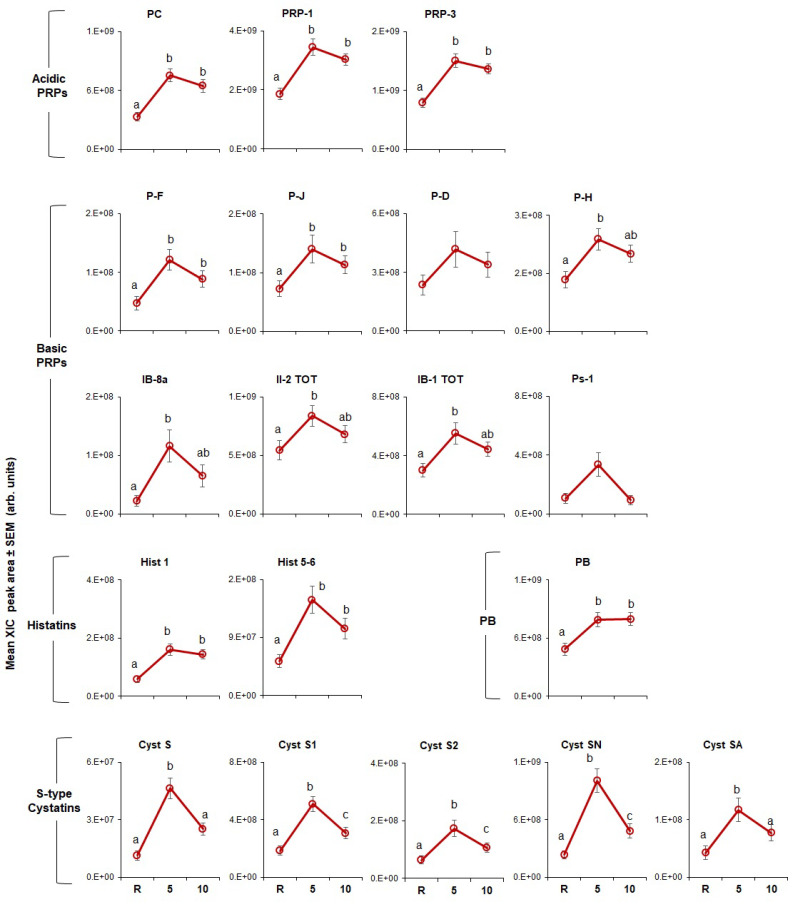
Effect of stimulation with CJ on levels of individual salivary proteins. Mean extracted ion current (XIC) peak areas ± SEM (×10^8^ arbitrary units) of individual proteins measured at resting (R), 5 min (5) and 10 min (10) after stimulation (*n* = 60). Means within protein type with different superscript letters (a, b, etc.) are different at *p* < 0.002.

**Figure 5:**
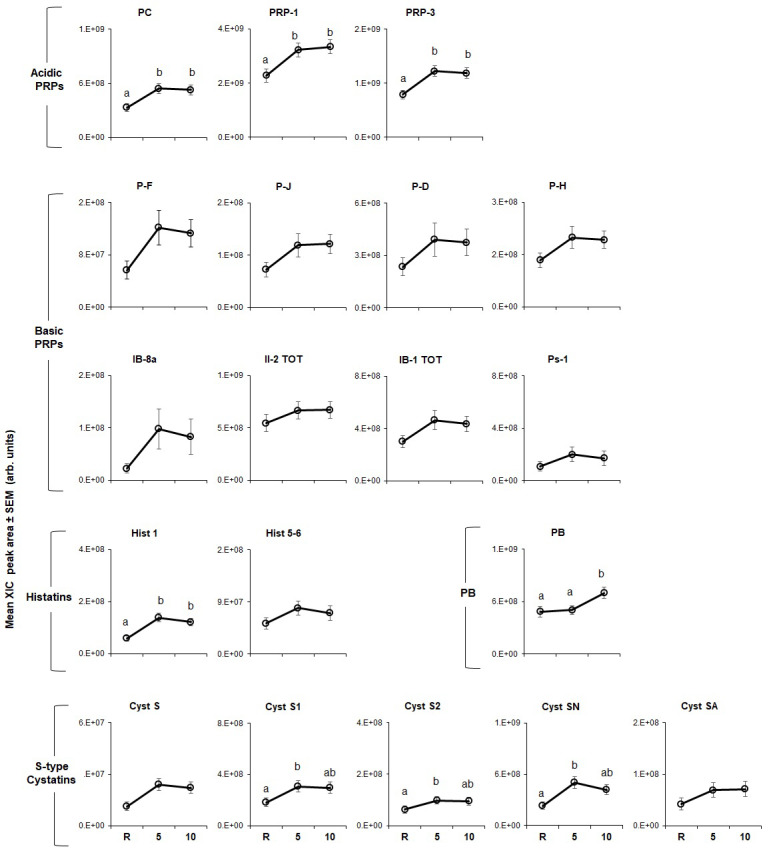
Effect of stimulation with CPE on levels of individual salivary proteins. Mean extracted ion current (XIC) peak area ± SEM (×10^8^ arbitrary units) of individual proteins measured at resting (R), 5 min (5) and 10 min (10) after stimulation (*n* = 60). Means within protein type with different superscript letters (a, b) are different at *p* < 0.002.

The effects of CJ and CPE stimulation on the levels of individual proteins are shown in Figure 4; Figure 5, respectively. In general, results showed that both stimuli led to increased levels of these proteins.

CJ stimulation (Figure 4) had a significant effect on all proteins (*p* = 0.002–0.0001) except for PD and Ps-1 (bPRP family). P-C, PRP-1, PRP-3 (aPRP family), PF and PJ (bPRP family) rose after stimulation with CJ at 5 min and remained elevated after 10 min. The same pattern was observed for Hist 1 and Hist 5, and Hist 6 (histatin family) and PB. Figure 4 also shows that, PH, IB-8a, II-2 and IB-1 (bPRP family), Cyst S and Cyst SA (S-type cystatins family) were also elevated at 5 min but fell to levels not statistically different from resting by 10 min. Three members of the S-type cystatins family, Cyst S1, S2, SN rose to peak levels at 5 min, trended downward, but remained elevated relative to baseline at 10 min.

The effects of CPE (Figure 5) on individual proteins were less robust than they were for CJ (*p* < 0.002). Levels of all three aPRPs were higher at 5 min and remained elevated at 10 min after stimulation with CPE; the same effect was observed for Hist 1 (histatin family). Levels of Cyst S1, S2 and SN were also elevated at 5 min but fell to baseline by 10 min. In contrast, Hist 5, and Hist 6, Cyst S and Cyst SA and all bPRPs were unaffected by stimulation.

#### 3.1.3. Alpha Amylase

**Figure 6:**
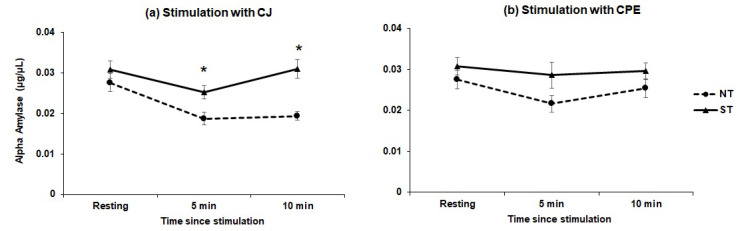
Effect of stimulation on alpha-amylase levels (µg/µL) after stimulation with CJ (**a**) and CPE (**b**). * indicates a difference between non-tasters and super-tasters (*p* < 0.014–0.000) at a given time point. Non-tasters (*n* = 29) and super-tasters (*n* = 31).

Mean alpha-amylase levels in resting saliva were estimated at 0.029 μg/uL. Repeated measures ANOVA showed no overall main effect of stimulation by CJ or CPE on alpha-amylase levels. However, there was a significant taster*treatment interaction, in response to stimulation (*F*_[4,224]_ = 5.95; *p* = 0.001). As shown in Figure 6, PROP STs had higher levels of amylase than NTs at 5 (*p* < 0.014) and 10 min (*p* < 0.000) after exposure to CJ. No effect of stimulation was seen with CPE.

Finally, there was no effect of stimulus presentation order on levels of alpha-amylase over the time course (*p* = 0.762, not significant).

## 4. Discussion

The time course of astringency has been studied from a sensory perspective [1,30,31] but complementary work in salivary proteins has been limited [33] thus far. The first objective of this study was to examine salivary responses to CJ and CPE with respect to major protein families and individual protein sub-types within those families. First, at the family level (Figure 2), we found that oral stimulation with CJ and CPE led to a robust increase in aPRPs but not the other protein families, which only showed minor (non-significant) positive increases. This generally agrees with our previous findings [52] with the same set of proteins showing that aPRPs rose robustly after oral stimulation with CJ. Another time-course study conducted by Brandao and co-workers [33] also reported that aPRPs were most responsive to stimulation with condensed tannins, while bPRP levels were relatively unaffected. Interestingly, we observed the same outcomes for aPRPs as did Brandao et al. [33] even though our study was fundamentally different than the earlier study, which had a smaller sample size (triplicates of *n* = 4), used pooled saliva samples and repeated oral stimulation prior to beginning the time course. Despite these methodological differences, all three studies support an important role for aPRPs in the salivary response to oral polyphenols. Together, these findings seem to conflict with the earlier literature suggesting that bPRPs were primarily responsible for oral astringency due to their highest binding affinity to tannins [14,70]. However, recent competitive in vitro assays showed that aPRPs and histatins, mostly found in the mobile phase of saliva, are first in line to interact with polyphenols (in comparison to other proteins such as mucins or gPRPs, which are mostly adsorbed onto mucosal or dental surfaces) particularly at lower phenolic concentrations [71]. Presumably, all the astringent stimulus may not be cleared from the mouth upon swallowing. It is possible that interaction of protein with residual polyphenols in saliva may occur [42,44], leading to persistent soluble aggregate formation, which do not coalesce and precipitate, initiating the so-called ‘second step’ of protein–polyphenol interaction [13,14].

An important observation in our study was that CJ was a more potent stimulus for protein responses than CPE, both at the family level (Figure 2) as well as for individual protein sub-types (Figure 4 and Figure 5). Virtually all protein sub-types were significantly elevated by CJ, except two bPRP sub-types, PD and Ps-1. In comparison, CPE elevated all three aPRPs, but its effects on other protein types were more muted and less consistent than what we observed for CJ. CPE did not increase any of the individual bPRPs and only affected some of the S-type cystatins and histatins. Finally, we noticed that individual S-type cystatins, (e.g., Cyst S1, S2 and SN) returned to baseline at the end of 10 min after CJ but not after CPE.

There could be several explanations for these differences. First, CPE is a carrier-free flavor ingredient that contains a mixture of various flavanoids. A preponderance of these polyphenols are anthocyanins, flavanols and proanthocyanidins, which have all been shown to interact with salivary proteins [24,25,72]. Although CJ contains these same polyphenols, it is also highly acidic and contains pectin. Pectins have been shown to hinder the complex formation between salivary proteins and polyphenols [23]. Acidic stimuli elicit saliva release, mainly from the parotid gland, which leads to an elevated level of proteins, such as proline-rich proteins in the oral cavity [73,74]. Notably, bPRPs are only released from the parotid gland, where they make up 23% of the total protein secreted [75]. This could be an explanation for why bPRP levels rose after CJ but not to CPE stimulation. Second, recent work studying mixtures of proteins in vitro has shown that depending on the size of protein–polyphenol aggregates formed by other salivary proteins such as aPRPs, interactions between phenolic stimuli and bPRPs may be impeded [25]. It is possible that CPE formed aggregates with other salivary proteins, which prevented the interaction between bPRPs and CPE. Finally, a large component of our understanding of protein–polyphenol interactions comes from purified salivary protein fractions, whereas the present study analyzed whole saliva, which is a mixture of many proteins. These factors alone or in combination could have influenced the higher levels of proteins following stimulation with CJ in contrast to CPE.

The present study also examined the role of PROP taster status in salivary protein responses to CJ with the goal of replicating the earlier findings of Melis and co-workers [52] and potentially extending these findings to CPE. Melis et al. [52] showed PROP-specific effects of CJ on two sub-types of aPRPs (PRP-1 and PRP-3) and one of the Cystatin sub-types (Cyst-SN). Specifically, levels of these proteins rose after CJ stimulation in medium-tasters and super-tasters, but no increases were observed in non-tasters. The present findings diverge from our earlier results in that here, we observed no PROP-related effects on either aPRP or Cystatin sub-types. However, we did observe a taster by gender interaction of stimulation on the bPRP protein family (Figure 3) only in STs. Generally, levels of bPRPs among male STs rose significantly higher than those of female STs at 5 min after stimulation. CPE stimulation also raised bPRPs for male STs but these levels were not significantly higher than those of female STs. The reasons for the discrepant findings between studies are presently unclear. Nevertheless, an important difference is that Melis et al. [52] measured protein levels at 1 min after stimulation, whereas the current study examined protein levels at 5 and 10 min. The dominance of different salivary proteins at different time points (i.e., aPRPs and cystatins at 1 min and bPRPs at 5 min after stimulation) suggests the sequential involvement of proteins at different stages of astringency development and that PROP-taster status may be an important marker at each stage of this process. Although methodologically challenging, repeated saliva collections at short intervals directly following CJ exposure may reveal important dynamics of the early protein response that would clarify our understanding of PROP effects as well as other individual differences in salivary function. Future studies should address this question. Interestingly, Melis et al. [52] also found a gender dichotomy in their astringency perception experiment. Together, these data suggest a gender-specific role for PROP status that may link oral astringency perception with protein responses. Greater recognition of these potential differences may be important for interpreting the results of future sensory studies and proteomic analyses.

To our knowledge, salivary amylase has not been measured in studies investigating the involvement of salivary proteins in astringency responses over a time course. We found no main effect of either CJ or CPE on amylase levels. However, a novel finding of this study was that STs had higher levels of amylase in response to CJ exposure than did NTs (Figure 6). Salivary amylase plays a key role in the oral digestion of starches by hydrolyzing complex carbohydrates to smaller sugars [76] and its expression has been shown to be influenced by high-fat and high-tannin diets in animal models [77,78]. Individual differences in salivary amylase activity are well known and may be related to *AMY1* gene copy number [79] in humans. Whether salivary amylase activity also varies with PROP taster status should be investigated in future studies as amylase has important implications in digestion, nutrient absorption and most recently, texture perception [80,81,82,83].

Seminal work by Dinnella and colleagues [42,44] on individual variation in the astringency response utilized total protein concentrations (*D* values) in correlation with sensory responses to classify individuals into low or high responders to astringency. In the current study, we targeted specific proteins and peptides, which enabled us to offer insights into a selected individual difference, i.e., interindividual variation in salivary proteins associated with PROP taster status. Conducting a time-course of astringent sensations in PROP-classified subjects could supplement our understanding of the sensory relevance of these differences. Examples of this could include use of temporal dominance of sensations or other time-intensity measures to track differences in the experience of astringent sub-qualities, which may be important for understanding this complex sensation.

The present study has several limitations. First, we did not measure salivary flow rate which has been correlated with measures of astringency perception in previous studies [19,42,84,85]. This decision was based on previous observations (Melis et al., unpublished) [86] showing no meaningful associations between flow rate and protein variations. Nevertheless, salivary flow has also been shown to be associated with differences in gender and age [87], particularly in the context of stimulation with sour stimuli [88]. Other work has shown that there can be considerable inter-individual variation in salivary flow rate and other characteristics related to diet and physiological factors [89,90] which could have impacted the variables measured in this study. Second, we did not assess mucins, which are technically challenging to measure [91]. Mucins play a key role in oral lubricity and including them in future studies will provide a more complete picture of astringency mechanisms. Third, in designing this study we weighed the potential impacts of presenting the two stimuli in different sessions on different days, each with its own baseline (potentially increasing session-to-session variability) or presenting them sequentially in a single session with a long rest period in between samples. We ultimately decided on the second scenario in which sample presentation was randomized to minimize order effects, and a 20 min rest period in between samples. In exploratory analyses we probed the data for order effects (i.e., the effects of subjects receiving CJ first vs. CPE first) but were unable to detect these differences. However, it is not known if the 20 min rest period was sufficient to reset protein levels to baseline. Finally, we did not collect saliva samples in replicates, i.e., participants were exposed to each stimulus only once. It is possible that there could be variation in the findings such as between-day variability if replications were performed. Nevertheless, whether small, underlying differences influenced our data is not known and should be considered in interpreting our findings.

## 5. Conclusions

These data show that proteomic changes may linger even at 10 min after an astringent high-polyphenol food enters the oral cavity. Therefore, researchers are cautioned against utilizing shorter breaks when administering astringent stimuli in sensory studies to allow the mouth sufficient time to recover. The present study used a time-course approach to study interindividual differences in salivary protein response to astringent stimulation and has provided novel insights into how PROP taster status may influence variation in astringency perception, i.e., via differential involvement of specific proteins, which may ultimately guide selection of polyphenol-rich foods.

## Figures and Tables

**Table 1:** Subject Characteristics.

Gender	PROP Classification *	*n*	Ethnicity (*n*)		Age	BMI
			Caucasian	Asian	(Years)	(kg/m^2^)
**Female**	**NT**	15	12	3	21.7 ± 1.5	26.0 ± 1.5
	**ST**	16	12	4	23.3 ± 1.5	24.2 ± 1.5
**Male**	**NT**	14	9	5	21.4 ± 0.6	24.2 ± 1.0
	**ST**	15	10	5	21.6 ± 1.2	25.5 ± 1.0

* NT: Non-Tasters, ST: Super-Tasters.

## Supplementary Materials

The following are available online at https://www.mdpi.com/2072-6643/12/9/2878/s1. Table S1: List of salivary proteins and peptides quantified by HPLC-low resolution-ESI-MS.
