# Supplementary material for: Time Course of Salivary Protein Responses to Cranberry-Derived Polyphenol Exposure as a Function of PROP Taster Status
Source: Nutrients. 2020 Sep 21;12(9):2878. doi: 10.3390/nu12092878 (PMC7551352; doi:10.3390/nu12092878)
Supplement: Supplementary file 1 [file nutrients-12-02878-s001.pdf]

Supplementary Table S1

List of salivary proteins and peptides quantified by HPLC-low resolution-ESI-MS.

Protein (Swiss-Prot Code)*	Experimental Average Mass (Da) ± SD (Theoretical)	Elution Time (min ± 0.5)
Acidic proline-rich phosphoproteins family (aPRPs):		
**P-C peptide (P02810)**	4370.9 ± 0.4 (4370.8)	13.6-14.5
PRP-1 type di-phosphorylated (P02810)	15515 ± 2 (15514-15515)	22.9-23.3
PRP-1 type mono-phosphorylated	15435 ± 2 (15434-15435)	23.9-24.3
PRP-1 type non-phosphorylated	15355 ± 2 (15354-15355)	24.2-24.7
PRP-1 type tri-phosphorylated	15595 ± 2 (15594-15595)	22.6-22.9
PRP-3 type di-phosphorylated (P02810)	11161 ± 1 (11161-11162)	23.3-23.8
PRP-3 type mono-phosphorylated	11081 ± 1 (11081-11082)	23.8-24.2
PRP-3 type non-phosphorylated	11001 ± 1 (11001-11002)	24.8-25.1
PRP-3 type di-phosphorylated Des-Arg_{106}	11004 ± 1 (11005-11006)	23.5-23.8
Histatin family (Hists):		
Histatin-1(P015515)	4928.2 ± 0.5 (4928.2)	23.3-23.8
Histatin-1 non-phosphorylated	4848.2 ± 0.5 (4848.2)	23.4-23.8
Histatin-6 (P15516)	3192.4 ± 0.3 (3192.5)	14.0-14.4
Histatin-5 (P15516)	3036.5 ± 0.3 (3036.3)	14.2-14.7
Basic proline-rich protein family (bPRPs):		
Ps-1	23460 ± 3 (23459.0)	17.0-18.0
P-J	5943.9 ± 0.5 (5943.6)	14.1-14.7
P-H (P02812/P04280)	5590.2 ± 0.5 (5590.1)	15.0-15.5
P-F (P02812)	5843.0 ± 0.5 (5842.5)	14.3-14.8
P-D (P010163)	6949.5 ± 0.7 (6949.7)	15.2-15.8
II-2 (Tot):		
- II-2 (P04280)	7609 ± 1 (7609.2)	18.7-19.1
- II-2 non-phosphorylated	7529 ± 1 (7529.2)	19.5-19.8
- II-2 Des-Arg_{75}	7453 ± 1 (7453.0)	18.8-19.2
IB-8a (Tot):		
- IB-8a (Con1+)	11888 ± 2 (11887.8)	17.1-17.8
- IB-8a (Con1-)	11898 ± 2 (11896.2)	17.1-17.8
IB-1 (Tot):		
- IB-1 (P04281)	9593 ± 1 (9593.4)	18.8-19.3
- IB-1 non-phosphorylated	9513 ± 1 (9513.4)	19.4-19.7
- IB-1 Des-Arg_{96}	9437 ± 1 (9437.2)	19.0-19.4
Statherin family (Staths)		
Statherin di-phosphorylated (P02808)	5380.0 ± 0.5 (5379.7)	28.9-29.5
Statherin mono-phosphorylated	5299.9 ± 0.5 (5299.7)	28.7-29.1
Statherin non-phosphorylated (P02808)	5220.5 ± 0.5 (5219.7)	28.4-28.8
Cystatin family (S-type Cysts)		
Cyst S non-phosphorylated (P01036)	14,186 ± 2 (14185)	36.5-37.1

Cyst S mono-phosphorylated (S1)	14,266 ± 2 (14265)	36.6-37.1
Cyst S di-phosphorylated (S2)	14,346 ± 2 (14345)	36.8-37.2
Cyst SN (P01037)	14,312 ± 2 (14313)	34.8-35.2
Cyst SA (P09228)	14,347 ± 2 (14346)	38.4-38.9
PB	5972.9 ± 0.5 (5792.7)	29.4-30.5

*Identification was based on the chromatographic behavior and comparison of the experimental mass values with the theoretical ones reported in the Swiss-Prot Data Bank (http://us.expasy.org/tools).
